# Iron overload mediates cytarabine resistance in AML by inhibiting the TP53 signaling pathway: Iron overload mediates cytarabine resistance

**DOI:** 10.3724/abbs.2025027

**Published:** 2025-02-28

**Authors:** Yan Jia, Ling Li, Ying Li, Xunxun Zhu, Haiyan wang, Bin Xu, Qiuping Li, Hao Zhang

**Affiliations:** 1 Department of Hematology Affiliated Hospital of Jining Medical University Jining 272000 China; 2 Department of Clinical Medicine Jining Medical University Jining 272000 China

**Keywords:** acute myeloid leukemia, TP53, iron overload, TFR1

## Abstract

Currently, chemotherapy remains the primary treatment for acute myeloid leukemia (AML). Drug resistance in AML cells is a critical factor contributing to the failure of chemotherapy remission and subsequent relapse. Iron overload frequently occurs in AML patients because of hematopoietic suppression or supportive blood transfusion therapy. Previous studies have indicated that iron overload may promote the progression of AML; however, the underlying mechanisms remain unclear. Our results demonstrate that, compared with TP53-wild-type AML cells, TP53-mutant AML cells exhibit increased resistance to cytarabine-induced cytotoxicity. Moreover, reducing TP53 expression in wild-type AML cells diminishes their sensitivity to cytarabine. The TP53 signaling pathway is essential for mediating cytarabine-induced apoptosis in AML cells. In this study, an iron overload model in AML cells via the use of ferric citrate is constructed. Our data indicate that iron overload can suppress the TP53/BCL2/BAX signaling pathway, counteracting cytarabine-induced apoptosis. In TP53 wild-type AML cells, TFR1 participates in iron-mediated resistance to cytarabine by regulating the entry of iron into the cells. These findings provide a foundation for further exploration of the molecular mechanisms involved in AML resistance to cytarabine.

## Introduction

Acute myeloid leukemia (AML) is a malignant hematological disorder that arises from hematopoietic stem/progenitor cells in the bone marrow and has a relatively high incidence in adults [ 1– 3]. The cornerstone of clinical treatment for AML is chemotherapy, which aims primarily to eradicate tumor cells through the induction of apoptosis. However, the emergence of apoptosis resistance in these tumor cells, often resulting from prolonged exposure to chemotherapy, significantly undermines the success of AML remission and leads to subsequent relapse [ 4– 6] .

TP53, a critical transcription factor, is involved in essential biological processes such as cell cycle arrest and apoptosis and can be activated by various stressors, including DNA damage, carcinogens, and nutrient depletion [ 7– 9]. It is the most frequently mutated gene in human tumors, primarily through missense mutations in the coding region or complete deletions of the *TP53* gene [ 10, 11]. In acute myeloid leukemia (AML), TP53 mutations serve as predictors of resistance and recurrence, with mutations or loss closely linked to chemotherapy resistance, irrespective of age, karyotype, or other genetic markers [ 12– 14]. Although TP53 mutations occur in less than 10% of newly diagnosed AML patients, their incidence is 25% in elderly patients and 30% in patients with therapy-related AML/myelodysplastic syndrome (t-AML/MDS) [ 15– 18]. Cytarabine (Ara-C) has been the cornerstone of induction and consolidation therapy for AML since the 1960s [19]. Despite a high initial remission rate in newly diagnosed patients, more than 50% of those who achieve first complete remission are expected to relapse within three years [20]. The TP53 signaling pathway is pivotal for the cytotoxic effects of targeted therapies and chemotherapy in AML [ 21, 22]. Clinical studies by Welch *et al*. [23] revealed a correlation between TP53 mutations during AML chemotherapy and treatment outcomes, a finding subsequently corroborated by experiments from Chang *et al*. [24]. Notably, the TP53 signaling pathway mediates both the efficacy of anti-AML therapies and the development of resistance [25].

Iron is a crucial element in various cellular processes, including DNA synthesis, oxygen transport, and ATP production [26]. Owing to their rapid proliferation and metastasis, tumor cells demand more iron than normal cells do [27]. Clinical studies have shown that genes associated with iron metabolism are upregulated during both the early and late stages of tumor development, further increasing the iron requirements of cancer cells [ 28, 29]. In animal models, low-iron diets have been shown to delay tumor growth, underscoring the role of iron in tumor progression [30]. An imbalance in iron homeostasis is recognized as a metabolic hallmark of malignant tumor cells and is characterized by increased iron demand throughout the processes of tumor development, survival, proliferation, and metastasis [31]. Conversely, excessive iron can increase oxidative stress, resulting in damage to DNA, proteins, and lipids, which may lead to apoptosis, ferroptosis, and necrosis [32]. Zhou *et al*. [33] demonstrated that iron enhances the cytotoxic effects of chemotherapy by generating reactive oxygen species (ROS) through oxidative stress, thereby inhibiting the growth and metastasis of melanoma. The role of iron in tumor biology remains contentious and warrants further investigation. Notably, iron metabolism directly affects TP53 via heme, influencing the localization, stability, and function of the TP53 protein, which in turn regulates TP53 signaling [34]. This interaction was corroborated in the study by Calabreseden *et al*. [35], where the application of iron chelators to AML cells not only impaired mitochondrial function but also diminished the TP53 signaling pathway.

Hematopoietic suppression and transfusion therapy frequently leads to iron overload in patients with acute myeloid leukemia (AML). Clinical investigations by Franke *et al*. [36] indicated that iron overload negatively impacts survival prognosis in AML patients. This disrupts the bone marrow microenvironment, thereby promoting the progression of AML [37]. However, there is a paucity of research regarding the effects of iron overload on the efficacy of AML chemotherapy agents. This study aimed to explore the impact of iron overload on the action of the chemotherapy drug cytarabine in AML and elucidate the underlying mechanisms involved.

## Materials and Methods

### Cell lines and cell culture

TP53-mutant AML cell lines (Skm1/Thp1) and TP53 wild-type AML cell lines (MOLM-13/MV4-11) (purchased from Tongpai Company, Shanghai, China) were used in this study. Skm1, Thp1, MOLM-13 and MV4-11 cells were cultured in RPMI-1640 medium (Jiruo Company, Hangzhou, China) supplemented with 10% fetal bovine serum and 5% penicillin-streptomycin at 37°C in a humidified atmosphere with 5% CO _2_. The cells were seeded at a density of 2 × 10 ^5^ cells/mL. Different concentrations of cytarabine (Selleck, Shanghai, China) were used to kill AML cells. AML cell iron overload models were generated via the use of various concentrations of ferric citrate (Sellseck).

### Real-time quantitative polymerase chain reaction (qPCR)

Total RNA was extracted using the RNeasy Mini kit (Qiagen, Hilden, Germany), and cDNA was synthesized using the PrimeScript RT reagent kit (TaKaRa, Dalian, China). Both procedures were carried out according to the manufacturers’ protocols. Total RNA (< 1 μg) from each sample was used to synthesize complementary cDNA. The RT reaction was performed at 37°C for 15 min, followed by 85°C for 5 s. Quantitative polymerase chain reaction (qPCR) was conducted via real-time PCR Master Mix (TaKaRa) on an ABI7500 real-time PCR system (Applied Biosystems, Foster City, USA). For amplification, an initial denaturation step at 95°C for 30 s was followed by 40 cycles at 95°C for 5 s, 60°C for 15 s, and 60°C for 34 s. The relative gene expression levels were calculated via the 2 ^–∆∆Cq^ method. *β-Actin* served as an endogenous control. The sequences of primers are as follows: *TP53* forward, 5′-CAGCACATGACGGAGGTTGT-3′/reverse, 5′-TCATCCAAATACTCCACACGC-3′; *BCL2* forward, 5′-GTGCGTGGAAAGCGTAGACA-3′/reverse, 5′-ATTCAGGTAAGTGGCCATCCAA-3′; *BAX* forward, 5′-CCTGTGCACCAAGGTGCCGGAACT-3′/reverse, 5′-CCACCCTGGTCTTGGATCCAGCCC-3′; *TFR1* forward, 5′-ACCATTGTCATATACCCGGTTCA-3′/reverse, 5′-CAATAGCCCAAGTAGCCAATCAT-3′ and *β-actin* forward, 5′-CCTTCCTGGGCATGGAGTCCTG-3′/reverse, 5′-GGAGCAATGATCTTGATCTTC-3′.

### Western blot analysis

The cells were lysed on ice for 10‒20 min in RIPA buffer (Gibco, Carlsbad, USA). The cell lysates were then centrifuged (12000 *g*), and the supernatants were collected for western blot analysis. Equal amounts of protein were separated by SDS-PAGE and blotted onto PVDF membranes (Millipore, Billerica, USA). The membranes were initially incubated with primary antibodies, including anti-TFR1 antibody (ab269513; Abcam, Cambridge, UK), anti-TP53 antibody (60283-2-Ig; Proteintech, Wuhan, China), anti-BCL2 antibody (66799-1-Ig; Proteintech), and anti-BAX antibody (60267-1-Ig; Proteintech), followed by incubation with the corresponding HRP-conjugated goat anti-mouse IgG(H+L) secondary antibodies (SA00001-1; Proteintech) for 1 h. Specific bands were visualized via an enhanced chemiluminescence (ECL) western blotting detection kit (Amersham Biosciences, Buckinghamshire, UK).

### Viral transfection assay

Sh-TP53/SH-TFR1 and TFR1-OE lentiviruses were purchased from Genechem Company (Shanghai, China). Transfection was performed according to the manufacturer’s protocol. The helper transfection reagent and the appropriate amount of virus were added to 2 × 10 ^6^/mL cells. The mixture was cultured at 37°C in a 5% carbon dioxide incubator for 8 h, after which fresh medium was added. After culture for 3‒4 days, western blot analysis and PCR were used to evaluate the transfection efficiency.

### Apoptosis assay

The apoptosis assay was performed via a 7-AADD/annexin kit (Multi Sciences Biotech Co., Ltd., Hangzhou, China). Flow cytometry was used to detect the percentage of apoptotic cells according to the manufacturer’s instructions. Annexin V/PE and 7-AADD were added to 100 μL of the cell suspension, which was then incubated for 15 min at room temperature (25°C) in the dark. The apoptotic cells were examined using a flow cytometer (BD, Franklin Lakes, USA).

### Cell viability assay

The cells were seeded in 96-well plates at a density of 2 × 10 ^5^ cells/mL, and the cell viability was determined after 48 h of culture via trypan blue staining. Briefly, the cell suspension was mixed with 0.4% trypan blue solution (Solarbio, Beijing, China) at a ratio of 9:1. After 3 min, the solution was dropped onto a hemocytometer. The average numbers of unstained cells and total cells were determined. The cell viability was determined as follows: cell viability = (average viable cell count/average total cell count) × 100%.

### Statistical analysis

Each experiment was repeated independently at least three times. Data are expressed as the mean ± standard deviation (SD). Statistical analysis was performed with SPSS 13.0 software (SPSS, Inc., Chicago, USA). The differences were analyzed with Student’s *t* test or one-way analysis of variance. For the cytotoxicity experiments, which included assessments of cell viability and apoptosis, each experimental group was repeated three times, and a *t* test was used to statistically analyze differences between groups. A *P* value less than 0.05 was considered to indicate a significant difference. Compusyn software was used to calculate the combined efficacy of iron and cytarabine: < 1 represents synergism, 1 represents additive, 1–10 represents antagonsim, and >10 represents very strong antagonsim.

## Results

### The TP53 signaling pathway is pivotal in mediating cytarabine-induced cytotoxicity

**Figure 1:**
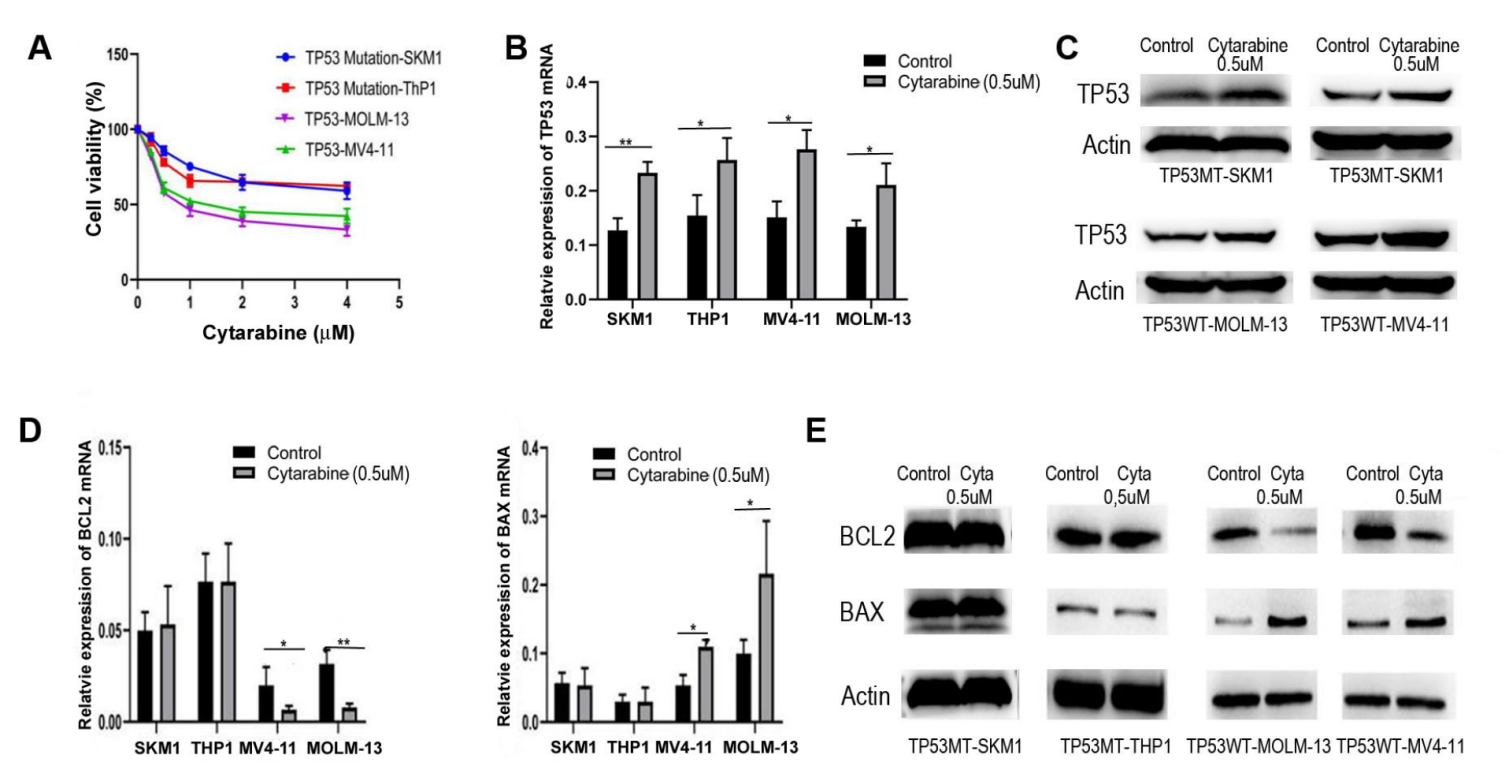
Figure 1 The TP53 signaling pathway plays a critical role in mediating cytarabine-induced cytotoxicity (A) TP53 mutant AML cells exhibited significantly reduced sensitivity to cytarabine compared to wild-type cells. (B,C) Following treatment with 0.5 μM cytarabine for 48 h, the expressions of TP53 signaling pathway components were analyzed by qPCR (B) and western blot analysis (C). Both TP53 wild-type and mutant AML cells showed significant upregulation of TP53 mRNA and protein levels post-treatment. (D,E) In TP53 wild-type AML cells, the expressions of downstream apoptotic targets, such as BAX, were markedly increased at both (D) mRNA and (E) protein levels, while BCL2 expression was significantly downregulated. In contrast, TP53-mutant AML cells showed no significant changes in the expression of BAX or BCL2. Data are presented as the mean ± standard deviations (SD) from at least three independent experiments. *P < 0.05, **P < 0.01, and ***P < 0.001.

Compared with those with TP53 mutations, AML cell lines with wild-type TP53 exhibit greater sensitivity to cytarabine. Specifically, AML cell lines, including TP53 wild-type (MOLM-13 and MV4-11) and TP53 mutant (SKM1 and THP1) cells, were treated with various concentrations of cytarabine (0, 0.25, 0.5, 1, 2, and 4 μM). Cell viability was assessed using the trypan blue exclusion method after a 24-h of incubation period. Compared with wild-type AML cells, mutant AML cells demonstrated significantly lower sensitivity to cytarabine ( Figure 1A). Following treatment with 0.5 μM cytarabine, the expression of TP53 signaling pathway components was analyzed using qPCR and western blot analysis after 48 h. Both TP53 wild-type and mutant AML cells presented significant increases in TP53 mRNA (SKM1: control vs 0.5 μM cytarabine, *P* < 0.01; THP1: control vs cytarabine, *P* < 0.05; MV4-11: control vs cytarabine, *P* < 0.05; and MOLM-13: control vs cytarabine, *P* < 0.05) and protein expression levels after treatment with cytarabine ( Figure 1B,C). In TP53 wild-type AML cells, the expression of downstream targets of the TP53 apoptotic signaling pathway, such as BAX, was significantly elevated at both the mRNA (MV4-11: control vs 0.5 μM cytarabine, *P* < 0.05; and MOLM-13: control vs 0.5 μM cytarabine, *P* < 0.05) and protein levels, whereas BCL2 expression was notably reduced (MV4-11: control vs 0.5 μM cytarabine, *P* < 0.05; and MOLM-13: control vs 0.5 μM cytarabine, *P* < 0.01) ( Figure 1D,E). In contrast, no significant alterations in the expression levels of BAX and BCL2 were detected in TP53-mutant AML cells ( Figure 1D,E). Although cytarabine induces TP53 expression in TP53-mutant AML cells, it does not affect the downstream molecules involved in TP53-mediated apoptosis. These findings indicate that cytarabine effectively activates the TP53 apoptotic signaling pathway in TP53 wild-type AML cells but has a minimal effect on TP53 mutant AML cells.

### TP53 is a key promoter of cytarabine-induced cytotoxicity in AML cells

**Figure 2:**
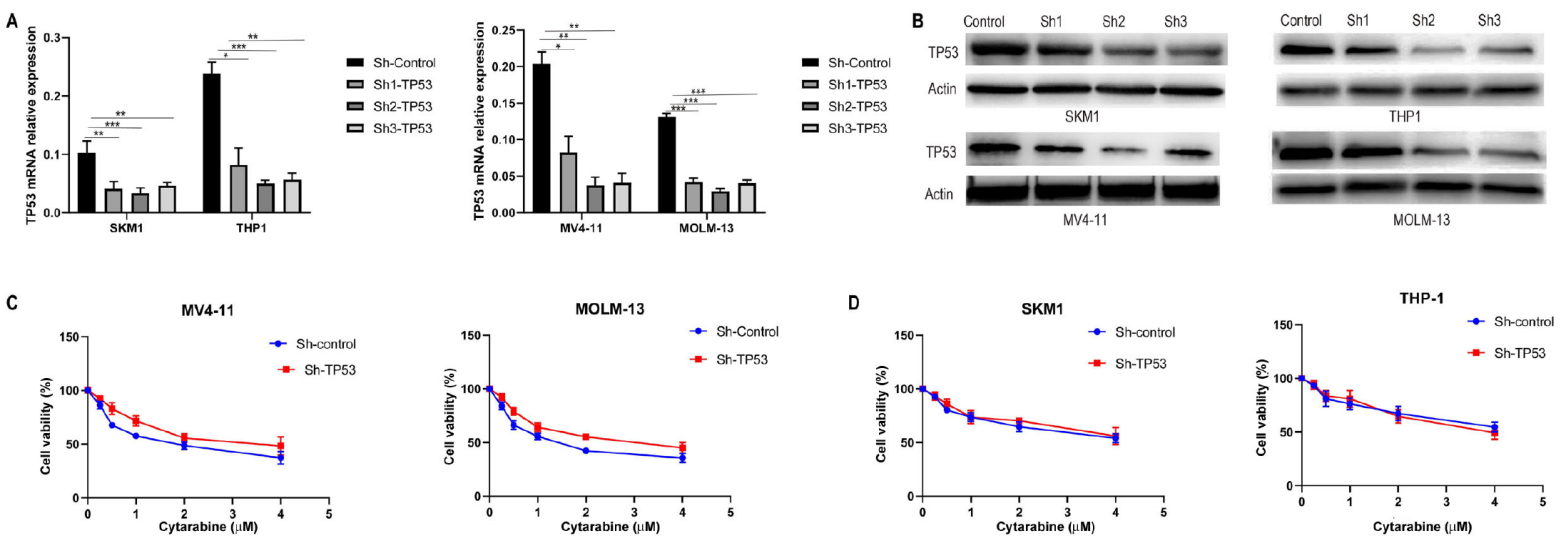
Figure 2 Iron overload suppresses the TP53 signaling pathway in wild-type TP53 AML cells (A) Low concentrations of ferric citrate enhanced the proliferation of wild-type TP53 AML cells, whereas high concentrations inhibited proliferation. In contrast, TP53-mutant AML cells showed no significant response to low concentrations of ferric citrate, but growth was suppressed at high concentrations. (B,C) Following TP53 knockout, the viability of wild-type TP53 AML cells increased with higher ferric citrate concentrations (B), while no significant changes were observed in TP53-mutant AML cells (C). (D,E) After 48 h of iron treatment (250 and 500 μM), TP53 mRNA levels remained unchanged; however, a significant reduction in TP53 protein expression was observed in both wild-type and mutant TP53 AML cells. Notably, in wild-type TP53 AML cells, BAX expression decreased, while BCL2 expression increased following ferric citrate treatment. No significant changes in BAX or BCL2 expression were detected in TP53-mutant AML cells. Data are presented as the mean ± standard deviations (SD) from at least three independent experiments. *P < 0.05, **P < 0.01, and ***P < 0.001.

To investigate its role, we employed a lentivirus to knock out the *TP53* gene in AML cell lines. The knockout efficiency was evaluated at 24 and 48 h post transduction using qPCR and western blot analysis. The results demonstrated that lentiviral constructs Sh1, Sh2, and Sh3 effectively knocked out the *TP53* gene, resulting in significantly reduced mRNA (SKM1: control vs Sh1, *P* < 0.01; control vs Sh2, *P* < 0.001; control vs Sh3, *P* < 0.01; THP1: control vs Sh1, *P* < 0.05; control vs Sh2, *P* < 0.001; control vs Sh3, *P* < 0.01; MV4-11: control vs Sh1, *P* < 0.05; control vs Sh2, *P* < 0.01; control vs Sh3, *P* < 0.01; MOLM-13: control vs Sh1, *P* < 0.001; control vs Sh2, *P* < 0.001; control vs Sh3, *P* < 0.001) and protein expression levels ( Figure 2A,B). Among these, Sh2 exhibited the highest efficacy and was selected for subsequent experiments. After *TP53* knockout, the cytotoxic effects of various concentrations of cytarabine (0, 0.25, 0.5, 1, 2, and 4 μM) were assessed after 24 h. The data indicated that *TP53* knockout in wild-type TP53 AML cells led to decreased sensitivity to cytarabine ( Figure 2C). Conversely, no significant difference in cytarabine sensitivity was observed in *TP53*-mutant cells ( Figure 2D).

### Iron overload inhibits the TP53 signaling pathway in wild-type TP53 AML cells

**Figure 3:**
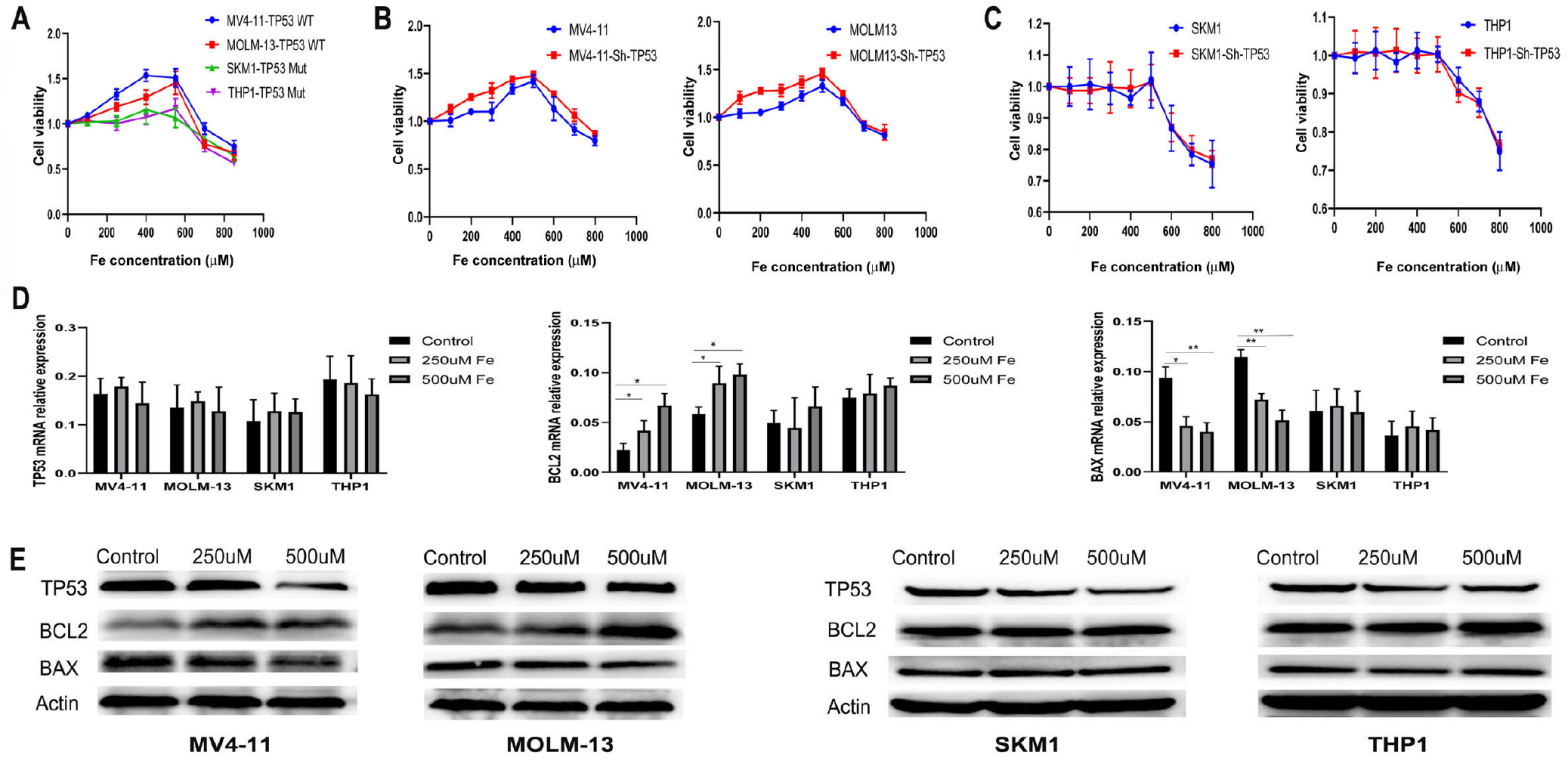
Figure 3 TP53 is a critical mediator of cytarabine-induced cytotoxicity in AML cells (A,B) Lentiviral constructs Sh1, Sh2, and Sh3 effectively knocked out the TP53 gene, leading to significant reductions in both mRNA and protein expression levels. (C,D) TP53 knockout in wild-type TP53 AML cells resulted in significantly reduced sensitivity to cytarabinen (C). In contrast, no notable change in cytarabine sensitivity was observed in TP53-mutant cells (D). Data are presented as the mean ± standard deviations (SD) from at least three independent experiments. *P < 0.05, **P < 0.01, and ***P < 0.001.

Within a specific concentration range, iron promotes the proliferation of wild-type TP53 AML cells without significantly affecting mutant TP53 AML cells. Various concentrations of ferric citrate (0, 125, 250, 500, 1000, and 2000 μM) were tested for their effects on AML cell viability and proliferation. After 48 h of treatment, the results of the trypan blue exclusion experiment indicated that low concentrations of ferric citrate promoted the proliferation of wild-type TP53 AML cells, whereas high concentrations inhibited it ( Figure 3A). In TP53-mutant AML cells, low concentrations had minimal effects, but high concentrations inhibited cell growth ( Figure 3A). Following *TP53* knockout, the viability of wild-type TP53 AML cells increased with increasing ferric citrate concentration ( Figure 3B), with no significant change observed in mutant TP53 AML cells ( Figure 3C). On the basis of these findings, a concentration of 500 μM ferric citrate was chosen for subsequent experiments. After 48 h of iron treatment (250 and 500 μM), *TP53* mRNA levels remained unchanged; however, a significant decrease in protein expression was noted in both wild-type and mutant TP53 AML cells ( Figure 3D,E). Notably, BAX expression (MV4-11: control vs 250 μM, *P* < 0.05; control vs 500 μM, *P* < 0.01; MOLM-13: control vs 250 μM, *P* < 0.01; control vs 500 μM, P<0.01) decreased, whereas BCL2 expression (MV4-11: control vs 250 μM, *P* < 0.05; control vs 500 μM, *P* < 0.05; MOLM-13: control vs 250 μM, *P* < 0.05; control vs 500 μM, *P* < 0.05) significantly increased in wild-type AML cells after ferric citrate treatment ( Figure 3D,E), with no changes observed in mutant TP53 AML cells. These results indicate that iron overload is involved in the modulation of the TP53 signaling pathway in wild-type TP53 AML cells.

### Iron overload enhances the resistance of wild-type AML cells to cytarabine

**Figure 4:**
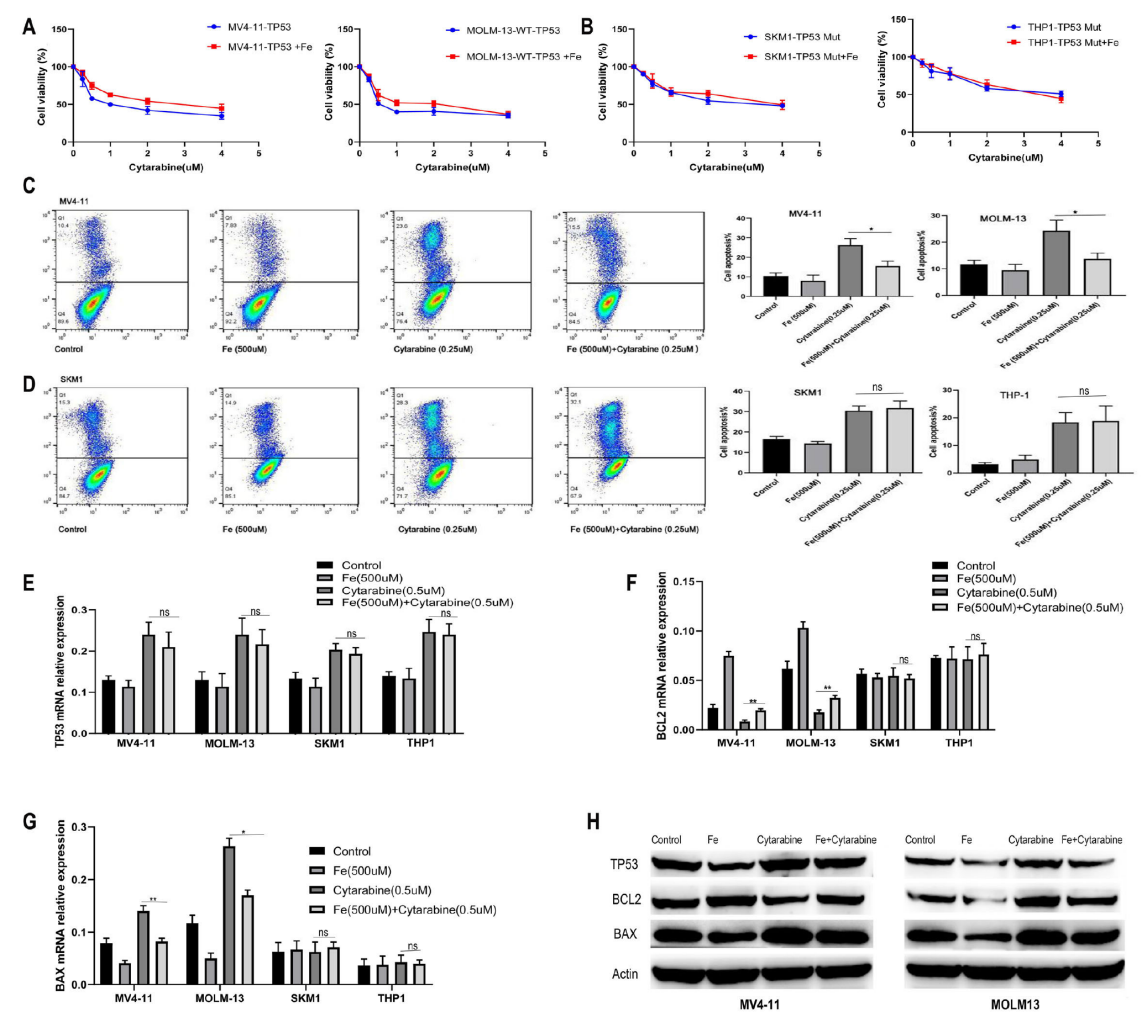
Figure 4 Iron Overload enhances the resistance of wild-type AML cells to cytarabine (A) The addition of ferric citrate significantly attenuated the cytotoxic effects of cytarabine in TP53-wild-type acute myeloid leukemia (AML) cells. (B) In contrast, no such effect was observed in TP53-mutant AML cells. (C,D) The addition of ferric citrate significantly reduced the cytotoxicity of cytarabine in wild-type AML cell lines. However, no significant effect was observed in TP53-mutant AML cell lines (THP-1 and SKM-1). (E) No significant change in TP53 mRNA expression was observed. (F,G) Ferric citrate mitigated the cytarabine-induced decrease in BCL2 mRNA in TP53-wild-type AML cell lines. (H) At the protein level, the addition of ferric citrate counteracted the cytarabine-induced upregulation of TP53 expression in both TP53-wild-type and TP53-mutant AML cell lines. In TP53-wild-type cells, ferric citrate reversed the cytarabine-mediated reduction in BCL2 expression and the increase in BAX expression, whereas no such effects were observed in TP53-mutant cells (4H). Data are presented as the mean ± standard deviations (SD) from at least three independent experiments. *P < 0.05, **P < 0.01, and ***P < 0.001.

The effects of 500 μM ferric citrate on the cytotoxicity of various concentrations of cytarabine (0, 0.25, 0.5, 1, 2, and 4 μM) were assessed after 24 h. The results demonstrated that the addition of iron significantly reduced the cytotoxic effect of cytarabine on TP53-wild-type AML cells ( Figure 4A). In contrast, iron did not affect the cytotoxicity of cytarabine in mutant AML cells ( Figure 4B). To further investigate this phenomenon, AML cells were treated with 500 μM of ferric citrate and 0.25 μM of cytarabine for 24 h, and the combined effects were assessed using flow cytometry. The results confirmed that ferric citrate significantly reduced the cytotoxic effect of cytarabine on wild-type AML cells (MV4-11/MOLM-13) (MV4-11: 0.25 μM cytarabine vs 0.25 μM cytarabine + 500 μM Fe, *P* < 0.05; MOLM-13: 0.25 μM cytarabine vs 0.25 μM cytarabine + 500 μM Fe, *P* < 0.05), whereas no significant effect was observed in mutant TP53 AML cells (THP-1/SKM1) ( Figure 4C,D). To further explore the effects of iron overload on the cytotoxicity of cytarabine, we utilized qPCR and western blot analysis to examine alterations in components of the TP53 signaling pathway. Following a 48-h treatment with 0.5 μM cytarabine and 500 μM ferric citrate, we measured the expression levels of TP53 signaling pathway molecules through qPCR and western blot analysis. Our results revealed no significant change in *TP53* mRNA expression ( Figure 4E). Nonetheless, the presence of 500 μM iron (ferric citrate) reduced the cytarabine-induced decrease in *BCL2* mRNA (MV4-11: 0.5 μM cytarabine vs 500 μM Fe + 0.5 μM cytarabine, *P* < 0.01; MOLM-13: 0.5 μM cytarabine vs 500 μM Fe + 0.5 μM cytarabine, *P* < 0.01) and the increase in *BAX* mRNA (MV4-11: 0.5 μM cytarabine vs 500 μM Fe + 0.5 μM cytarabine, *P* < 0.01; MOLM-13: 0.5 μM cytarabine vs 500 μM Fe + 0.5 μM cytarabine, *P* < 0.05) in TP53 wild-type AML cell lines ( Figure 4F,G). At the protein level, the addition of iron diminished the increase in TP53 expression induced by cytarabine, as observed in both the TP53 wild-type and mutant AML cell lines ( Figure 4H). In TP53 wild-type AML cell lines, iron reversed the reduction in BCL2 expression and the increase in BAX expression triggered by cytarabine; however, similar effects were not observed in TP53 mutant-type cells ( Figure 4H).

The influence of different concentrations of ferric citrate (0, 125, 250, and 500 μM) on the cytotoxic effects of different concentrations of cytarabine (0, 0.25, 0.5, 1, 2, and 4 μM) was examined. The combined effects were calculated via Compusyn software, and the results are shown in Table 1 and Table 2. The data show the antagonistic effects of different iron concentrations on various doses of cytarabine.

**Table:** **Table 1** Cytotoxic effects of different concentrations of iron against various concentrations of cytarabine in MV4-11 cells

Cytarabine dose (μM)	Fe dose (μM)	Effect	CI
0.25	125	0.125	3.2182
0.25	250	0.134	2.7592
0.25	500	0.155	4.87483
0.5	125	0.161	7.54715
0.5	250	0.171	3.1538
0.5	500	0.216	2.4215
1.0	125	0.226	1.5670
1.0	250	0.247	2.5428
1.0	500	0.284	4.5661
2.0	125	0.299	3.068
2.0	250	0.309	2.2536
2.0	500	0.412	3.0437
4.0	125	0.542	8.1962
4.0	250	0.552	1.1256
4.0	500	0.608	1.2568

**Table:** **Table 2** Cytotoxic effects of different concentrations of iron against various concentrations of cytarabine in MOLM-13 cells

Cytarabine dose (μM)	Fe dose (μM)	Effect	CI
0.25	125	0.174	2.34120
0.25	250	0.112	4.78648
0.25	500	0.162	2.62826
0.5	125	0.245	2.62304
0.5	250	0.145	6.26568
0.5	500	0.241	2.70065
1.0	125	0.359	2.52343
1.0	250	0.315	3.28703
1.0	500	0.311	3.37006
2.0	125	0.325	6.18112
2.0	250	0.184	17.0950
2.0	500	0.414	3.69622
4.0	125	0.449	6.10457
4.0	250	0.49	4.89475
4.0	500	0.771	0.91080

### Knocking out *TFR1* attenuates the antagonistic effect of iron overload on cytarabine-induced cytotoxicity in TP53-wild-type AML cells

**Figure 5:**
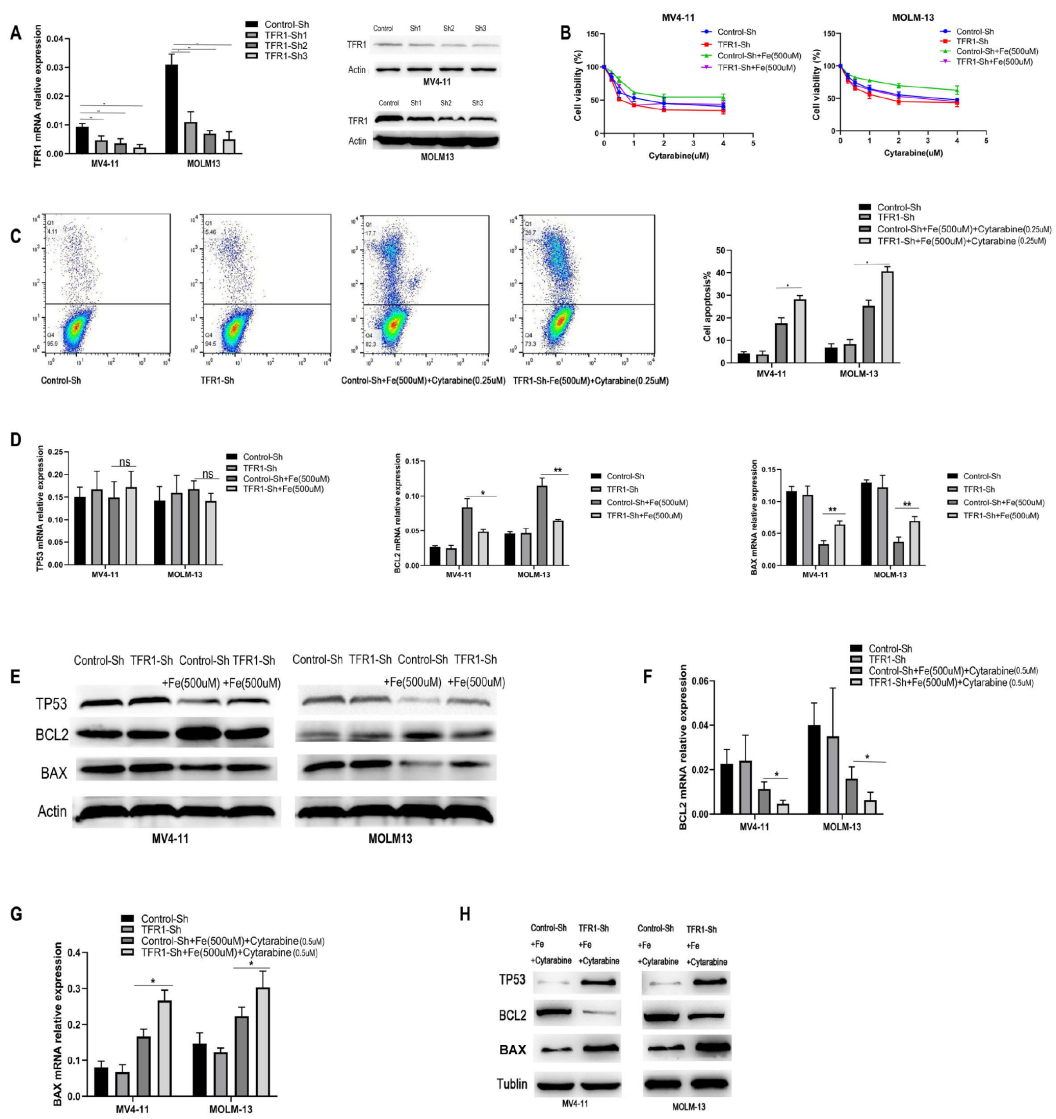
Figure 5 Knocking out *TFR1* attenuates the antagonistic effect of iron overload on cytarabine-induced cytotoxicity in TP53-wild-type AML cells (A) Three shRNA constructs (Sh1, Sh2, and Sh3) significantly reduced TFR1 mRNA and protein expression, with Sh3 exhibiting the most efficient knockdown ratio. (B) Downregulation of TFR1 expression attenuated the impact of iron on cytarabine-induced cytotoxicity across a range of cytarabine concentrations (0, 0.25, 0.5, 1, 2, and 4 μM) after 24 h of treatment. (C) Flow cytometry analysis revealed that reduced TFR1 expression diminished the protective effect of iron (500 μM) against cytarabine (0.25 μM)-induced cytotoxicity in TP53-wild-type AML cells. (D,E) TFR1 knockout did not significantly alter the expressions of TP53 signaling pathway components (TP53, BCL2, and BAX) compared to the control group. However, the addition of iron (500 μM ferric citrate) decreased TP53 and BAX expressions while increasing BCL2 expression in AML cells. In the TFR1 knockout groups, the effects of iron on TP53 signaling pathway molecules were significantly attenuated. (F-H) After TFR1 knockout and treatment with cytarabine (0.5 μM) and iron (500 μM ferric citrate), BAX mRNA and protein expressions were significantly higher in the TFR1 knockout group than in the control group. Conversely, BCL2 mRNA and protein expression were significantly lower in the TFR1 knockout group than in the control group. These findings suggest that TFR1 knockdown mitigates the impact of iron on cytarabine-induced cytotoxicity in TP53-wild-type AML cells. Data are presented as the mean ± standard deviations (SDs) from at least three independent experiments. * P < 0.05, **P < 0.01, and ***P < 0.001.

Transferrin receptor 1 (TFR1) is the primary receptor for iron entry into cells, and reducing its expression inhibits iron uptake. Our data indicate that knocking out *TFR1* diminishes the antagonistic effect of iron on cytarabine-induced cytotoxicity in AML cells. We used Sh-TFR1 virus transfection to downregulate TFR1 expression and assessed its mRNA levels via qPCR at 24 h and protein levels via western blot analysis after 48 h. All the Sh-TFR1 constructs (Sh1, Sh2, and Sh3) exhibited decreased mRNA (MV4-11: control vs Sh1, *P* < 0.01; control vs Sh2, *P* < 0.01; control vs Sh3, *P* < 0.01; MOLM-13: control vs Sh1, *P* < 0.05; control vs Sh2, *P* < 0.01; control vs Sh3, *P* < 0.01) and protein expressions, with Sh-3 demonstrating the most effective knockdown ( Figure 5A). Thus, Sh-3 was employed in subsequent experiments. Downregulated TFR1 expression in AML cells reduced the antagonistic effect of iron on the cytotoxicity induced by various concentrations of cytarabine (0, 0.25, 0.5, 1, 2, and 4 μM) after 24 h ( Figure 5B). Specifically, decreased TFR1 expression diminished the impact of 500 μM iron on the cytotoxicity induced by 0.25 μM cytarabine, as assessed using flow cytometry (MV4-11: control-+ 500 μM Fe + 0.25 μM cytarabine vs sh-TFR1 + 500 μM Fe + 0.25μM cytarabine, *P* < 0.05; MOLM-13: control + 500 μM Fe + 0.25 μM cytarabine, *P* < 0.05) ( Figure 5C). After knocking out *TFR1*, there were no significant differences in the expressions of TP53 signaling pathway molecules (TP53, BCL2, and BAX) compared with those in the control group ( Figure 5D,E). The addition of iron (500 μM ferric citrate) decreased the expression levels of TP53 and BAX while increasing BCL2 expression. In the *TFR1* knockout groups, the effect of iron on these signaling molecules was weakened ( Figure 5D,E). Furthermore, following *TFR1* knockout in AML cells treated with cytarabine (0.5 μM) and iron (500 μM), we observed that, in the *TFR1* knockout group, BAX expression (MV4-11: control + 500 μM Fe + 0.5 μM cytarabine vs sh-TFR1 + 500 μM Fe + 0.5 μM cytarabine, *P* < 0.05; MOLM-13: control + 500 μM Fe + 0.5 μM cytarabine vs sh-TFR1 + 500 μM Fe + 0.5 μM cytarabine, *P* < 0.05) was greater, and BCL2 expression (MV4-11: control + 500 μM Fe + 0.5 μM cytarabine vs sh-TFR1 + 500 μM Fe + 0.5 μM cytarabine, *P* < 0.05; MOLM-13: control + 500 μM Fe + 0.5 μM cytarabine vs sh-TFR1 + 500 μM Fe + 0.5 μM cytarabine, *P* < 0.05) was lower than that in the control group ( Figure 5F–H). These findings suggest that knocking down *TFR1* can reduce the impact of iron on cytarabine-induced cytotoxicity.

### Upregulating TFR1 expression in TP53-wild AML cells intensifies the antagonistic impact of iron on cytarabine

**Figure 6:**
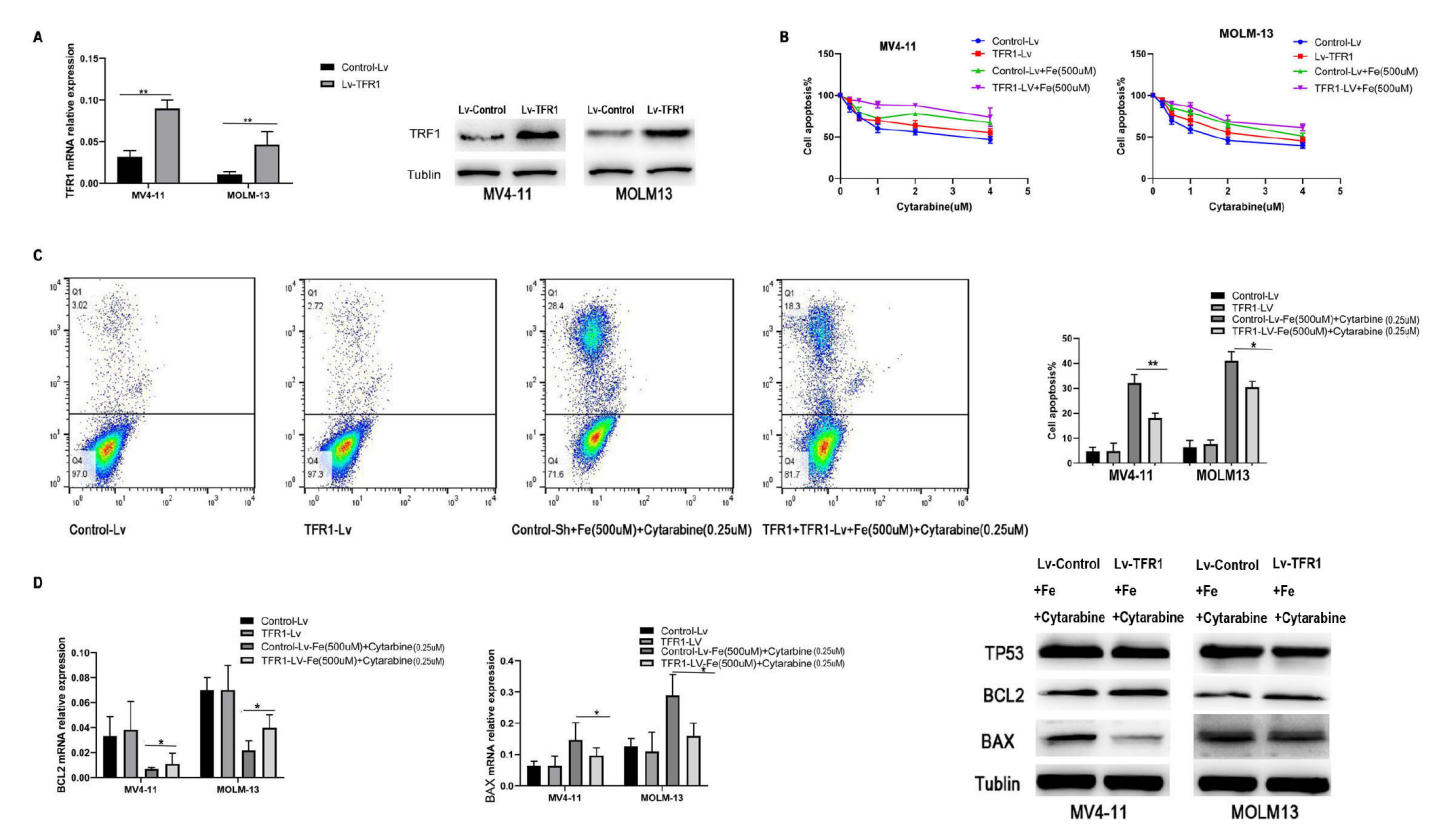
Figure 6 Elevated TFR1 expression enhances iron-mediated antagonism of cytarabine in TP53-wild-type AML cells (A) Following 72 h of LV-TFR1 transfection, significant increases in both mRNA and protein levels of TFR1 were observed. (B) While TFR1 overexpression alone did not significantly alter cytarabine sensitivity, the addition of iron markedly reduced cellular responsiveness to cytarabine. Notably, LV-TFR1-transfected cells treated with iron exhibited the lowest sensitivity to cytarabine. (C) Flow cytometry analysis further demonstrated that elevated TFR1 expression amplified the inhibitory effect of iron (500 μM) on cytarabine-induced cytotoxicity (0.25 μM). (D) TFR1 overexpression in AML cells treated with cytarabine (0.5 μM) and ferric citrate (500 μM) resulted in decreased BAX mRNA and protein levels, alongside increased BCL2 mRNA and protein expression levels, compared to controls. * P < 0.05, **P < 0.01, and ***P < 0.001.

To further investigate the effects of iron overload on the TP53 signaling pathway, we upregulated TFR1 expression in TP53-wild-type AML cells using viral transfection. Following LV-TFR1 virus transfection for 72 h, we observed a significant increase in both the mRNA (MV4-11: control vs LV-TFR, *P* < 0.01; MOLM-13: control-LV vs LV-TFR1, *P* < 0.01) and protein levels of TFR1 in AML cells ( Figure 6A). The overexpression of TFR1 in AML cells enhanced the antagonistic effect of iron on cytarabine. As shown in Figure 6B, after TFR1 overexpression, AML cells were treated with various concentrations of cytarabine (0, 0.25, 0.5, 1, 2, or 4 μM) for 24 h. The results indicated no significant changes in sensitivity to cytarabine alone; however, the addition of iron reduced the sensitivity of these cells to cytarabine. Notably, the LV-TFR1 with iron group presented the lowest sensitivity to cytarabine. Increased TFR1 expression increased the impact of iron (500 μM) on the cytotoxicity induced by cytarabine (0.25 μM) after 24 h, as assessed by flow cytometry (MV4-11: control + 500 μM Fe + 0.25 μM cytarabine vs TFR1-Lv + 500 μM Fe + 0.25 μM cytarabine, *P* < 0.01; MOLM-13: control- + 500 μM Fe + 0.25 μM cytarabine vs TFR1-Lv + 500 μM Fe + 0.25 μM cytarabine **,** *P* < 0.05) ( Figure 6C). Following TFR1 overexpression and treatment with cytarabine (0.5 μM) and iron (500 μM ferric citrate), we found that in the TFR1 overexpression group, BAX expression (MV4-11: control-control + 500 μM Fe + 0.25 μM cytarabine vs TFR1-Lv + 500 μM Fe + 0.25 μM cytarabine, *P* < 0.05; MOLM-13: control + 500 μM Fe + 0.25 μM cytarabine vs TFR1-Lv + 500 μM Fe + 0.25 μM cytarabine, *P* < 0.05) was decreased, whereas BCL2 expression (MV4-11: control + 500 μM Fe + 0.25 μM cytarabine vs TFR1-Lv + 500 μM Fe + 0.25 μM cytarabine, *P* < 0.05; MOLM-13: control + 500 μM Fe + 0.25 μM cytarabine vs TFR1-Lv + 500 μM Fe + 0.25 μM cytarabine, *P* < 0.05) was increased compared with that in the control group ( Figure 6D).

## Discussion

Patients with AML are prone to iron overload due to bone marrow suppression and transfusion therapy. Clinical investigations by Franke *et al*. [36] revealed that the survival prognosis is significantly worse for AML patients with iron overload than for those without iron overload. They also reported that iron overload negatively impacts overall survival rates during or prior to stem cell transplantation [36]. This disrupts the bone marrow microenvironment, which plays a crucial role in the progression of AML [37]. However, the specific mechanisms underlying these effects still need to be explored.

The primary focus of clinical cancer treatment is chemotherapy, which aims primarily at inducing apoptosis in tumor cells. However, prolonged use of chemotherapeutic drugs can lead to apoptosis resistance and drug resistance [ 4– 6]. The TP53 signaling pathway plays a crucial role in regulating cell apoptosis during chemotherapy-induced tumor cell death [ 21, 22]. Notably, TP53 mediates the resistance of AML cells to the chemical drug cytarabine [ 38, 39]. Our research indicates that wild-type TP53 AML cells are more sensitive to cytarabine-induced cytotoxicity than mutant TP53 AML cells. To further investigate the role of TP53 in cytarabine-induced cytotoxicity, we downregulated TP53 expression in wild-type TP53 AML cells via lentivirus. We found that decreased TP53 expression diminishes sensitivity to cytarabine, whereas decreasing TP53 expression in mutant AML cells does not significantly affect their response to the drug. Furthermore, in wild-type TP53 AML cells treated with cytarabine (0.5 μM), both TP53 and BAX expressions increased. In contrast, in mutant TP53 AML cells, only TP53 expression was altered, whereas the expressions of its downstream targets BCL2 and BAX remained unchanged. These findings suggest that cytarabine activates the TP53 signaling pathway in wild-type TP53 AML cells, highlighting its critical role in cytarabine-induced cytotoxicity.

Iron is essential for critical biological processes, including cellular DNA synthesis, and serves as an indispensable micronutrient for cells [26]. The rapid proliferation of tumor cells leads to an increased demand for iron [27]. Recent research has shown that iron-mediated ferroptosis can effectively eliminate tumor cells and prevent the development of chemotherapy resistance [40]. In our experiments, we observed that within a certain concentration range, iron promoted the proliferation of wild-type TP53 AML cells, whereas high concentrations of iron decreased cell viability. When TP53 expression was reduced in wild-type TP53 AML cells, cell viability increased in response to various concentrations of ferric citrate compared with that in controls; however, no significant effect was noted in mutant TP53 AML cells. Following the addition of ferric citrate to AML cells, we detected no change in TP53 mRNA expression. In contrast, a significant decrease in the protein level was observed in both wild-type and mutant TP53 AML cells, indicating that iron promotes a reduction in TP53 expression at the protein translation level rather than at the mRNA transcription level. Furthermore, in wild-type AML cells treated with ferric citrate, BAX expression decreased, whereas BCL2 expression increased. No differences were observed in the expression of BAX and BCL2 in mutant AML cells. Our results revealed that iron can enhance the activation of the TP53 signaling pathway in wild-type TP53 AML cells by regulating TP53 protein expression. Additionally, we found that iron antagonizes cytarabine-induced cytotoxicity in wild-type TP53 AML cells, whereas no significant effect of iron on cytarabine-induced cytotoxicity was observed in mutant AML cells. These findings suggest that iron overload may inhibit cytarabine-induced cytotoxicity in AML cells, possibly through the suppression of TP53 signaling pathway activity.

*TFR1* encodes a protein essential for cellular iron uptake, facilitating the transport of transferrin-bound iron into cells and playing a crucial role in maintaining iron homeostasis [41]. Cells with high proliferation rates and high energy demands, such as cancer cells, osteoclasts, and activated lymphocytes, present increased iron requirements, resulting in elevated TFR1 expression [ 42, 43]. The rapid proliferation of tumor cells and their increased iron demand lead to the overexpression of TFR1, which enhances iron absorption [44]. Consequently, TFR1 is considered a potential target for cancer therapy. In our experiments, we used lentiviral methods to knock out *TFR1*, which resulted in a decrease in the iron antagonism of cytarabine-induced cytotoxicity in AML cells. Conversely, overexpressing TFR1 via lentivirus increased the iron antagonism of cytarabine-induced cytotoxicity in these cells. These findings suggest that further investigation into the role of iron metabolism in mediating cytarabine-induced cytotoxicity is warranted.

Iron overload promotes the progression of AML and is closely related to patient survival. These experimental results strongly support the notion that iron overload antagonizes cytarabine-induced cytotoxicity in AML cells, possibly through the inhibition of the TP53 signaling pathway. This study provides a theoretical basis for understanding the mechanism of cytarabine resistance in AML treatment and offers new insights for treating AML.
